# Surgical correction of hyperlordosis in facioscapulohumeral muscular dystrophy: A case report

**DOI:** 10.1186/s12893-017-0276-0

**Published:** 2017-07-17

**Authors:** Haining Tan, Fan Feng, Youxi Lin, Chong Chen, Zheng Li, Jianxiong Shen

**Affiliations:** Department of Orthopedics, Peking Union Medical College Hospital and Graduate School of Peking Union Medical College, Peking Union Medical College, Chinese Academy of Medical Science, Beijing, China

**Keywords:** Facioscapulohumeral muscular dystrophy, Hyperlordosis, Scoliosis, Wheelchair-dependent, Surgical correction

## Abstract

**Background:**

Hyperlordosis is common in facioscapulohumeral muscular dystrophy (FSHD), which cannot be controlled by bracing. While the surgical treatment is neither reported nor recommended in previous studies, we report the first corrective surgery for hyperlordosis in one wheelchair-dependent FSHD patient.

**Case presentation:**

A 15-year-old, wheelchair-dependent girl complaining of hyperlordosis and lower extremity weakness was diagnosed as FSHD. Preoperative examination showed hyperlordosis of 116° with scoliosis of 44°. Posterior correction and bone graft fusion was performed. Postoperatively, the hyperlordosis was corrected to 72° and better sitting posture was gotten. 12 months’ follow-up showed favorable correction with improvement in SF-36 and ODI score.

**Conclusions:**

The correction for hyperlordosis in FSHD is controversial. We report the first successful case of operative treatment by corrective spine surgery in these rare and demanding patient collective. Corrective surgery for spinal deformity, such as hyperlordosis and scoliosis, could do some help in posture and improve the quality of life especially in wheelchair-dependent patients.

## Background

Facioscapulohumeral muscular dystrophy (FSHD) is one of the most common forms of muscular dystrophy with prevalence of 1/20000 to 12/100000, characterized by weakness of facial, shoulder-girdle and humeral muscles [1]. Deletion of a polymorphic repeat D4Z4 on chromosome 4q35 and mutation in *structural maintenance of chromosome flexible hinge domain containing 1 (SMCHD1)* are correlated with FSHD [2, 3]. Although FSHD progresses slowly, about 20%–36.9% patients need the wheelchair during the rest of their life. Unfortunately, there is no effective pharmacologic therapy for FSHD, but regular aerobic exercise may improve fitness [4].

Hyperlordosis is common in FSHD, which results in weakness of pelvic extensor and paraspinal muscles [5, 6]. Bracing for hyperlordosis is ineffective to control progression. However, corrective surgery has not been reported and even not recommended in patients from previous studies [5, 7].

We report the first corrective surgery for hyperlordosis in one wheelchair-dependent FSHD patient, who shows improvement in posture, Medical Outcomes Study Short Form-36 (SF-36), and Oswestry Disability Index (ODI) postoperatively, indicating that correction for hyperlordosis can help to improve posture and quality of life for wheelchair-dependent FSHD patients.

## Case presentation

**Fig. 1:**
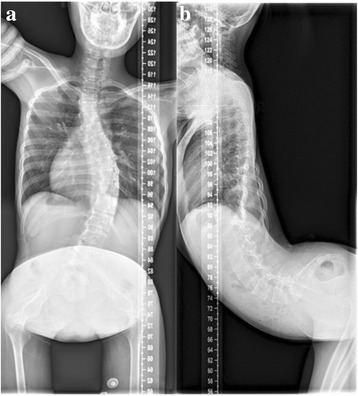
Preoperative anterior-posterior and lateral X-ray of whole spine. **a** Anterior-posterior X-ray showed Cobb of scoliosis (T5-L2) is 44°. **b** Lateral X-ray showed Cobb of thoracolumbar lordosis (T9-S1) is 116°

**Fig. 2:**
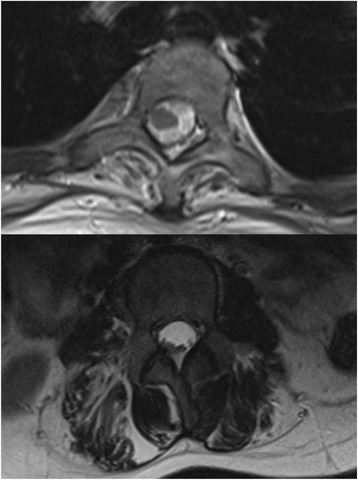
Magnetic resonance imaging (MRI) showed wasting and fat infiltration in paraspinal muscles

A 15-year-old girl was admitted into our spine center in November 2015 with complaint of gradually aggravated hyperlordosis for 4 years and lower extremities weakness for 1 year. She had been wearing the brace for more than 4 years (20 h/day) without controlling the hyperlordosis. She was dependent in the wheelchair and couldn’t stand without any support. Medical history included pectus excavatum at birth, and corrective operation was performed 4 years ago. Physical examination found weakness of facial expression muscles (bilateral inability to completely close the eyes, frown, bulge the cheek, purse the lips, and whistle), winged scapula of both sides and decreased strength in back, proximal upper and lower extremities (grade II-III on the Oxford scale). The Gower sign was positive due to the weakness of abdominal and iliopsoas muscles, while her intelligence, vision and hearing acuity were all normal. Her mother also had weakness of expression muscles without spinal deformity. Anterior-posterior (AP) and lateral standing X-ray of whole spine revealed thoracolumbar hyperlordosis (T9-S1, 116°), scoliosis (T5-L2, 44°), and horizontally positioned sacrum (Fig. 1). Computed tomography (CT) showed similar deformities. Wasting and fat infiltration in paraspinal muscles was detected from Magnetic resonance imaging (MRI), without any spinal cord or canal abnormality (Fig. 2). The electromyography (EMG) showed myogenic changes in proximal upper and lower extremity. The Creatine kinase MB (CKMB) was 5.8 μg/L, slight higher than the normal level (3.6 μg/L), but the Creatine kinase (CK) was normal (116 U/mL) as well as other laboratory tests.

**Fig. 3:**
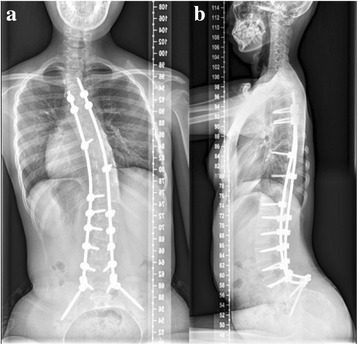
Anterior-posterior and lateral X-ray at 12-month follow-up. **a** Anterior-posterior X-ray showed the Cobb of scoliosis (T5-L2) is 29°. **b** Lateral X-ray showed Cobb of thoracolumbar lordosis (T9-S1) is 70°

FSHD was diagnosed on the basis of classic clinical presentations [8]. In December 2015, posterior internal fixation and bone graft fusion from T4-ilium was performed and the paraspinal muscle was obtained for biopsy. Postoperatively, the hyperlordosis and scoliosis were corrected to 72°and 35° separately. The appearance of leaning forward was improved obviously. The biopsy showed myogenic change and predominance of type I myofibril. The girl had been followed-up for 12 months with favorable correction for hyperlordosis and sitting posture (Fig. 3). The score of SF-36 increased from 124 to 526.5, while the score of ODI decreased from 66.7 to 51.1.

## Discussion

Hyperlordosis is common in FSHD, which results in weakness of pelvic extensor and paraspinal muscles [5, 6]. The fat infiltration, correlated with decreased muscle strength, can be detected in MRI, indicating the involvement of muscles in FSHD [9]. Previous study showed that bracing was ineffective to control the progression of hyperlordosis. But corrective surgery is not introduced, even thought to do more harms than benefits to patients who are ambulant [5]. Lee reported an 11-year-old girl diagnosed as early-onset FSHD with hyperlordosis similar with our patient, except for standing and walking independently. They thought the relatively strong back extensor muscles made the spine hyperextended to maintain upright posture, and gave chance to stand and walk. Thus, the corrective for hyperlordosis wasn’t recommended due to the possible damage to compensatory mechanism by back extensor muscles.

Unlike the patient reported by Lee, the back extensor muscles of this girl are weaker due to the deterioration of FSHD, and fail in maintaining compensatory mechanism. So, the girl is wheelchair-dependent and in bent posture when sitting. The involvement of paraspinal and pelvic extensors muscles in MRI can prove this. It can be expected that, hyperlordosis and bent posture will be worse and worse with weakness of paraspinal muscles, which has obvious influence on the daily life. So, the correction is necessary because the rigid internal fixation system could hold the upright sitting posture, while the paraspinal muscles were weak.

The changes in SF-36 and ODI scores can also prove the improvement in quality of life after correction of hyperlordosis.

## Conclusion

FSHD is one of the most common forms of muscular dystrophy, and many patients accompanies with spinal deformity including hyperlordosis or scoliosis. The spinal deformity influences the posture, even ability of movement. The correction for hyperlordosis in FSHD is controversial. We report the first successful case of operative treatment by corrective spine surgery in these rare and demanding patient collective. Corrective surgery for spinal deformity, such as hyperlordosis and scoliosis, could do some help in posture and improve the quality of life especially in wheelchair-dependent patients.

## Abbreviations

AP: Anterior-posterior
CK: Creatine kinase
CKMB: Creatine kinase MB
CT: Computed tomography
EMG: Electromyography
FSHD: Facioscapulohumeral muscular dystrophy
MRI: Magnetic resonance imaging
ODI: Oswestry disability index
SF-36: Medical outcomes study short form-36
SMCHD1: Structural maintenance of chromosome flexible hinge domain containing 1
